# Translational new approaches for investigating mood disorders in rodents and what they may reveal about the underlying neurobiology of major depressive disorder

**DOI:** 10.1098/rstb.2017.0036

**Published:** 2018-01-29

**Authors:** Emma S. J. Robinson

**Affiliations:** School of Physiology, Pharmacology and Neuroscience, Biomedical Sciences Building, University Walk, Bristol BS8 1TD, UK

**Keywords:** major depressive disorder, affective bias, animal model, neuropsychology, neurotrophic

## Abstract

Mood disorders represent one of society's most costly and challenging health burdens. The drug treatments used today were initially discovered serendipitously in the 1950s. Animal models were then developed based on the ability of these drugs to alter specific behaviours. These models have played a major role in the development of the second generation of antidepressants. However, their use has been heavily criticized, particularly in relation to whether they recapitulate similar underlying biology to the psychiatric disorder they are proposed to represent. This article considers our work in the field of affective bias and the development of a translational research programme to try to develop and validate better animal models. We discuss whether the new data that have arisen from these studies support an alternative perspective on the underlying neurobiological processes that lead to major depressive disorder (MDD). Specifically, this article will consider whether a neuropsychological mechanism involving affective biases plays a causal role in the development of MDD and its associated emotional and behavioural symptoms. These animal studies also raise the possibility that neuropsychological mechanisms involving affective biases are a precursor to, rather than a consequence of, the neurotrophic changes linked to MDD.

This article is part of a discussion meeting issue ‘Of mice and mental health: facilitating dialogue between basic and clinical neuroscientists’.

## 1. Introduction

Affective disorders are the most prevalent mental health conditions affecting modern society, with major depressive disorders (MDD) expected to become the leading cause of disability adjust life years by 2020. Emotional dysfunction and symptoms such as depression and anxiety are also highly co-morbid with other clinical conditions, particularly in chronic illnesses, such as chronic pain, addiction and neurodegenerative disorders. Other mental health conditions such as schizophrenia and bipolar disorder include emotional symptoms where negative affect and blunted emotions occur either as part of the disease or because of the medications used. Drug-induced anxiety, depression and/or suicidal ideation and behaviour are also a major challenge for the pharmaceutical industry [1]. For example, the cannabinoid_1_ (CB1) receptor family and its associated antagonists showed promise as novel treatments for obesity. However, evidence of increased risk of psychiatric side effects saw the withdrawal of rimonabant (a CB_1_ inverse agonist) shortly after it was licensed [2]. Other drug classes have also been linked with increased risk of psychiatric symptoms including the anti-acne medication, Roaccutane, and the immune-mediated treatment for hepatitis C, interferon alpha [3,4]. This diversity of clinical scenarios further illustrates the complexity of the problem and the challenges researchers face when trying to elucidate their cause(s) and develop effective treatments.

One of the primary challenges for all mental health conditions is being able to understand the underlying neurobiological processes that contribute to the disease. All drugs currently used in the treatment of psychiatric disorders were initially discovered because of clinical observations of novel agents, often being tested for entirely different indications. The detailed pharmacology of the drugs was not understood until many years after they were first used therapeutically [5]. As the field of psychopharmacology developed, a much better understanding of the individual receptor targets and biochemical effects of these drugs has been characterized. A very important outcome of this was the development of more selective drugs that could achieve similar biochemical effects but with a reduced side effect burden and improved safety. The development of the serotonin specific re-uptake inhibitors provides an excellent example of this. These drugs are now the most widely used treatments for both anxiety and MDD and are often prescribed to other patient populations to try to address co-morbid mood symptoms.

Although significant progress has been made in the development of better antidepressant drugs in terms of side effects and safety, knowledge about how their biochemical effects translate into improvement in mood remains limited. A major limiting factor has been the relationship between basic and clinical research. MDD is a disease characterized by a broad range of symptoms that are largely defined based on subjective self-report measures (e.g. DSM-V [6]). These criteria cannot be replicated directly in a pre-clinical scenario and as such, current animal models are assessed based on criteria such as *face* (resembles some characteristic of the human condition e.g. anhedonia, behavioural despair), *construct* (arises as a consequence of similar predisposing factors e.g. stress, genetic vulnerability, early life adversity) and *predictive* validity (the ability to predict in an animal the clinical effects of a treatment) [7–9]. No animal model for MDD has yet been developed that has achieved all three of these validation criteria. There is also a problem with poor translation between animal research and clinical benefits, with few new pre-clinical drugs being successfully taken forward to licensing. To try to address this, we have taken a novel approach building on developments in objective measures of emotional dysfunction in the clinical and experimental medicine fields. This article summarizes progress to date and considers the possible implications of the findings that have arisen from our validation work and investigations into novel neurobiology.

## 2. Limitations of current animal models

Previous authors have considered this issue in detail and as such, this section will only discuss animal models of depression briefly and in the context of the work presented here. The discussion also only considers methods used to test for depression-like behaviours and has not considered the methods used to induce a depression-like phenotype. For more detailed reviews of animal models used in psychiatry or depression research see [7,10–19].

Modelling human psychiatric disorders in animals is always going to present a challenge as researchers try to align subjective self-report measures of emotional disorders with a behavioural output in an animal, usually a rodent. The very nature of the attempts to relate animal to human behaviour leads to inevitable anthropomorphisms and the reality is that it is impossible to prove or disprove whether these behaviours are analogous. The classic behavioural tests in rodents are the forced swim test (FST) (rats) and tail suspension test (TST) (mice). These were originally developed to test for and predict antidepressant efficacy in the clinic [20,21]. Validation of these tests came from the ability of known antidepressant drugs to modify escape behaviour in animals in a way that was not seen with other psychoactive compounds (although Porsolt and others have warned of the potential confounds with locomotor stimulants) [13,22,23]. The tests have been very useful in the development of the second-generation antidepressants, however, use in phenotypic studies and research into underlying neurobiology are more controversial. Although the FST/TST have some face validity in that the animals exhibit behavioural despair in response to an inescapable stressor, how well this aligns with human MDD is less clear. In a recent review, Commons *et al.* [19] proposes that the FST and TST are measures of stress coping. Furthermore, the time course of effects has always been problematic. For example, both conventional delay-onset antidepressants and the rapid onset antidepressant, ketamine, have effects in the FST/TST following acute administration, something that is not seen in the clinic.

An alternative approach for assessing a behavioural phenotype relevant to depression has been the use of the sucrose preference test (or in some studies ICSS (intracerebral self-stimulation threshold) [18,24,25]). These tests are designed to measure an animal's hedonic response and to detect the development of an anhedonic phenotype. It has certainly been the case that chronic stress leads to a reduction in sucrose preference and this can be reversed by antidepressant treatments [26]. However, evidence of this type of hedonic deficit in patients is less robust. Loss of pleasure in daily activities is a core diagnostic feature in MDD. However, the human sweet taste test, which attempts to measure in humans a form of sucrose preference, failed to find a deficit in patients [27]. It is not clear whether the form of anhedonia seen in depressed patients is the same as the consummatory deficit recorded in animals, or more complex and linked with reward anticipation and expectation [28]. Recently, studies into depression-like phenotypes in rodents, particularly mice, have used the novelty supressed feeding test (NSFT) [29]. This test takes advantage of the natural aversion of rodents to novel environments and the suppression in feeding behaviour that this stress response induces. Generally considered to be more of a test of anxiety-related behaviour, the NSFT is sensitive to the anxiolytic effects of chronic but not acute antidepressants [30].

Whatever our concerns about animal models and depression research, animal studies are an essential part of fundamental biology and drug development. There remains a large gap in our knowledge that can only really be investigated using a combination of basic research and clinical investigations. Patient studies are important but often limited for practical and ethical reasons. Animal studies provide a much simpler system for testing hypotheses but this can only then feed back into the clinic if the methods used in the animals have translational validity [9]. Translating between humans and animals cannot be achieved when the human measure is made using language-based tasks or self-report measures. It is also not possible to expect an animal model to recapitulate symptoms such as suicidal ideation or low mood. However, there are well characterized neuropsychological deficits in MDD that do not depend on self-report measures [31]. The approach we have taken is to look at the neuropsychological deficits observed in MDD using computerized test batteries, or other objective methods, and then develop rodent tasks that we predict will depend on similar underlying neurobiology.

## 3. Affective biases in major depressive disorder: a neuropsychological biomarker?

Affective biases describe how emotional states can influence higher cognitive processes. The term has been most commonly used to describe biases in attention and emotional interpretation but similar biases are also proposed to modulate other cognitive domains including learning and memory and decision-making [31–40]. We have therefore taken a broad interpretation of the term ‘affective biases’ and suggest that this term is relevant across different cognitive domains where the emotional state of the organism leads to an altered response [31]. This is important when we consider the relationship between the human measures of affective biases and the approaches we have used in our animal work, particularly given that the emotional stimuli do not readily translate. In our consideration of affective biases, we have looked more widely at the deficits reported in MDD and used this evidence to inform our subsequent animal work (also see discussion by [41]).

While affective biases are not in themselves necessarily a pathological process, evidence would suggest that negative biases are a central feature of MDD. The question now is whether these biases develop because of the disease or in themselves contribute to its development and maintenance. Beck first proposed that a cognitive mechanism involving negative styles of thinking and interpretation played a critical role in the development and maintenance of mood disorders [42,43]. While Beck did not specifically focus on the neurobiology or neuropsychology of affective bias, his theories provide a useful foundation for the current ideas around affective bias and MDD. We have recently reviewed this topic [31] and therefore this section will provide only a brief synopsis with an emphasis on key areas relevant to the animal work and the development of novel behavioural tasks of affective bias in rodents.

In MDD, patients and at-risk populations tend to exhibit negative biases in their processing of emotional information, including biases in interpretation, memory and recall [32,34,44–49]. The most commonly reported affective biases in MDD include a reduced ability to recognize happy faces and increased sensitivity to negative emotions such as sadness and fear [35]. Although not involving a computerized testing method, studies looking at autobiographical memory suggest that negative biases may also affect the patient's perception of their environment and relationships as well as their associated memories [33,48,50,51]. The most important breakthrough in this area in terms of developing animal models has been the work by Harmer and colleagues [52–55] (also see this issue). Their work first suggested that antidepressant drugs could interact with neuropsychological processes to modify emotional interpretation. Not only did this link the drugs' effects with a relevant neuropsychological process, but the group also observed these effects following acute administration, in stark contrast to previous studies suggesting delayed onset. Healthy volunteers or patients were shown to respond to acute treatment with either a noradrenaline re-uptake inhibitor, reboxetine or serotonin specific re-uptake inhibitor, citalopram [52,53]. The participants did not report any subjective changes in mood, suggesting that these objective shifts in emotional processing could occur without conscious awareness [54,55]). The same group have also shown that emotional memory is similarly biased by acute antidepressant treatments [54,55]. Overall, this work suggests that negative biases in cognitive processes could provide a ‘neuropsychological biomarker’ for MDD. These biases are sensitive to acute modulation with antidepressant treatments that may predict longer term efficacy. For animal researchers, these tasks also provide a valuable starting point for developing new models to study depression-related behaviours.

## 4. The development and validation of rodent tasks of affective bias

**Figure 1.**
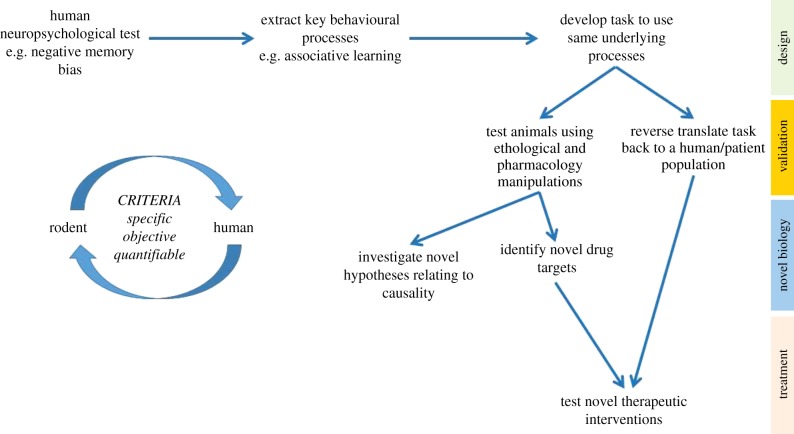
Schematic representation of a strategy for the development and validation of novel, translational behavioural methods for translational psychiatry. The choice of behavioural measure is key and should be specific to the condition of interest, objective and quantifiable. The process of developing the task and validation is illustrated through to the ideal scenario whereby the resulting behavioural approach can be across species to investigate novel biology, identify and develop new therapeutics and ultimately, test these in the same behavioural test in both pre-clinical and clinical drug development.

Our research has focused on two different cognitive domains: learning and memory and decision-making. These cognitive domains lend themselves more favourably to being studied in animals and, although not the same as the human tasks based on emotional stimuli, we suggest that they still measure behaviours relevant to the wider discussions surrounding affective biases, cognition and MDD. To study affective biases in relation to learning and memory, we developed the affective bias test (ABT) [56,57]. This task uses associative learning between a specific cue and reward to test the influence of affective state at the time of learning on the subsequent relative valuation of that reward. This task is different from reward learning tasks such as probabilistic learning [58–60] as it looks at the animal's memory of the experience, and relative value of the reward attributed to it, rather than the ability to learn about the current reward opportunities and adapt their behaviour accordingly. The judgement bias task (JBT) is designed to test affective biases linked to decision-making behaviour and tests animals' interpretation of ambiguous information within the context of positive versus negative or less positive associations. This task was originally designed by animal welfare researchers to facilitate their objective assessment of affective state in non-human species [61] but has proved to be a landmark study in terms of the wider field of affective biases. Aspects of the JBT may be like other tasks where animals' responses to reward and punishment are measured i.e. probabilistic learning tasks [60], however, the primary objective of the model is not to observe changes in responses to the reference cues but to see a specific shift in interpretation of an ambiguous cue. The general strategy we have used from the design of the task to deliver novel biology is outlined in figure 1. This is still a work in progress but ultimately may provide a new platform for novel biology and drug development. The development of new human tasks based on the reverse translation back to humans could also help provide tasks for clinical trials.

Developing tests for affective biases in rodents required a shift from studies in humans that use emotional stimuli e.g. faces or words, to cues that were relevant to other species. Animals lack language and while they may use facial cues and body language to communicate emotional information, this is unlikely to provide a realistic task for laboratory research. The first example was published by Harding *et al.* [61] in a study in rats. This task was based on human tasks that used ambiguous stimuli to probe affective state with evidence that negative mood states were strongly associated with pessimistic interpretation under ambiguity. The task involved training animals to associate previously neutral cues (in this case, auditory tones) with an emotionally valenced outcome (reward versus avoidance of punishment). Once the association was learnt, the animal's interpretation of ambiguous cues was tested by presenting an intermediate frequency cue and then looking at the animal's choice of response. Their work showed that animals in a putative negative affective state were less likely to anticipate reward in the same way that depressed people are more pessimistic. A more detailed discussion of this and related work is reviewed by Hales & Robinson [16]. A variety of different versions of this task have now been used, including high versus low/no reward, reward versus punishment avoidance and operant or spatial tasks [62–65]. The work provided the first empirical evidence that rodents possessed the neuropsychological capacity to express affective biases, in this case referred to as a *cognitive affective bias*. Similar judgement bias tasks (JBT) have now been tested in a wide range of species from flies to humans with similar findings (for review see Hales & Robinson [16]).

Our own work with the JBT has focused on pharmacological and ethological validation [64,65]. We first adapted the original task to include active choice for both the positive and negative/less positive outcomes to reduce potential confounds associated with motivational changes. Similar tasks have also reported by Enkel *et al*. [62] and Rygala's group [63,66–68]. We have tested a range of acute pharmacological treatments in both a high versus low reward version of the task and a reward versus punishment task with similar results [63,64]. Unlike the human emotional interpretation studies, we failed to observe any effects with acute antidepressant drug treatments [65]. Drugs that caused either anxiety or a stress response induced a negative bias, with animals becoming more pessimistic following treatment [62,63,65]. One study has reported a positive effect with acute doses of citalopram and the same study, as well as our own, found positive biases following treatment with amphetamine but not cocaine [65,68]. Chronic antidepressant treatment does induce a positive bias but the effect develops slowly over time [64,65]. However, ketamine behaves very differently and we have recently shown that an acute dose of ketamine but not phencyclidine (PCP) can induce more optimistic decisions in this task [69]. These findings seem to suggest that the effects of antidepressant drugs on decision-making behaviour in this rodent task occur over a timescale that more closely reflects the subjective self-report outcomes of treatment as opposed to the objective effects on emotional processing. The pharmacological data obtained so far do not show similar effects, or time course of effects, for the antidepressants tested, suggesting that decision-making in this rodent task involves different underlying neurobiology from the human emotional interpretation tasks. This may be because the animal task uses learnt associations between the cue and the affective outcome whereas the human tasks use stimuli that trigger innate responses. The rodent JBT may also be influenced by the prolonged training procedures required to teach animals the task, which can result in responding based more on procedural learning. Although the lack of concurrence with the human literature is disappointing, the differential effects seen for delayed versus rapid onset antidepressants are interesting and suggest this task may be useful for predicting the efficacy and time course of novel antidepressants. We have tried reverse translation of this rodent task for use in humans [70] and observed a correlation between anxiety and pessimistic behaviour in healthy volunteers. However, further studies using pharmacological treatments and in patients with depression are needed.

Patients with depression attribute less value to rewarding experiences than non-depressed people, particularly when they are considering past experiences [33,48,50,51]. Imaging studies also suggest blunted responses to rewarding stimuli and cues predicting reward [71–73]. We hypothesized that affective biases may modify learning and memory for rewarding experiences and that this could be measured in an animal task. In the ABT, an animal is given two independent learning experiences and then asked ‘which do you prefer?’ during a preference test. The learning we use is the association between a specific digging substrate (the cue) and a fixed value food reward (45 mg rodent reward pellet). The pairing sessions are carried out on different days and involve discrimination learning, with the animal deemed to have learnt the association when it can discriminate between the reward-baited substrate versus a non-rewarded substrate over six consecutive correct trials. Each reward association is made on a different day to ensure independence. This also means that we can manipulate conditions before one of the learning experiences. Following two pairing sessions for each condition (over 4 consecutive days), the animals are then presented with both of the previously rewarded substrates during a preference test. The test is carried out with random reinforcement to maintain responding but reduce any new learning. The resulting choice bias score is then calculated to determine if the treatment has induced a positive or negative bias. Thus, the idea behind the task is that the animal will re-activate its memory for the reward associated with each substrate and then bias its responding based on the relative value it attributes to each experience.

**Figure 2.**
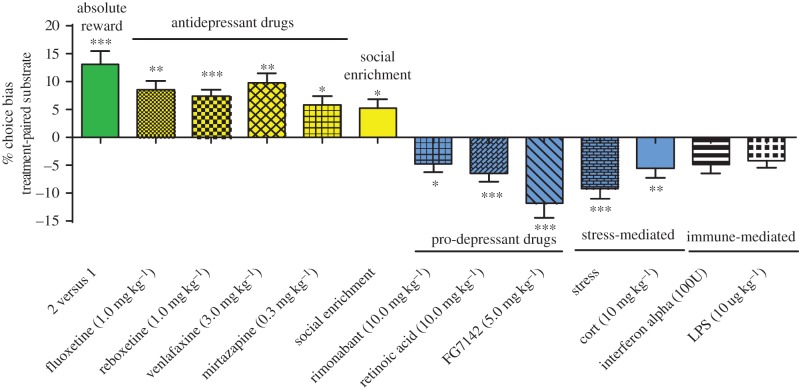
Summary of the pharmacological and psychosocial manipulations tested in the validation of the affective bias test (ABT). The results show that changes in absolute reward value as well as antidepressant drugs and social enrichment induce positive biases following acute treatment. In contrast, risk factors linked to the development of MDD in humans cause negative biases in this task. Adapted from [56,74,75]. LPS, lipopolysaccharide.

Initial proof of concept for the study design was achieved by testing whether changing the absolute value of the reward for each substrate–reward association would result in a positive bias towards the substrate associated with the higher value reward (figure 2). Once these proof of concept data were obtained, we progressed to testing a range of pharmacological interventions that have either antidepressant or pro-depressant effects in humans. We also tested the effects of psychosocial manipulations of affective state and drugs that have effects on the immune system (figure 2). Manipulations tested that induced a negative bias following acute treatment have all been linked to causing mood-related impairments in humans. We also find that antidepressants from a range of different classes induce a positive bias but neither drugs of abuse nor the failed antidepressant and neurokinin1 (NK1) antagonist, aprepitant, had a significant effect [56]. The work published in 2013 also showed that the bias could be observed irrespective of whether the treatment was given before or immediately after learning, suggesting a more complex integration of affective information with the substrate–reward association. We also observed that the bias increased with each successive pairing session [57]. We speculate that this may involve longer term memory consolidation processes. In a more recent series of experiments, we attempted to link the animals' performance in the ABT with neural circuits implicated in MDD, namely the amygdala and medial prefrontal cortex (mPFC). We also used the ABT to investigate whether the temporal differences in efficacy observed with delayed versus rapid onset antidepressants involved different interactions with this neuropsychological mechanism. In this study, we observed that biases linked to new learning were mediated through the amygdala, as lesions to this structure prevented the formation of a positive bias to venlafaxine and attenuated negative biases induced either pharmacologically or through stress [57]. The rapid onset antidepressant, ketamine, failed to have any effect on new learning so we tested whether pre-treatment with ketamine before the preference test could modify a previously learnt negative bias. We hypothesized that ketamine may mediate its rapid effects through an ability to block the negative affective bias associated with a previous memory. This was found to be the case as ketamine but not the conventional antidepressant, venlafaxine, was able to block negative biases induced by pharmacological or stress manipulations [57]. We were also able to localize this effect to the mPFC and found that inactivation of this brain area induced a similar attenuation. Importantly, this work has linked the ABT to a neural circuit implicated in MDD. Imaging studies suggest that dysfunctional activity in both the subgenual cingulate and amygdala are observed in MDD and studies suggest that remediation of this dysfunction corresponds with antidepressant efficacy [76]. This circuit may also be the target for deep brain stimulation [36,77].

Providing robust validation of an animal model for psychiatry research is not straightforward but is critical if the animal model is going to yield research outcomes with clinical relevance. The current methods used for MDD research have only limited validation with the evidence that both the FST and TST can produce false positive and false negative findings. The time course of effects for delayed versus rapid onset antidepressants in these tests is also problematic as both can induce changes in immobility time following acute treatment, something that is not observed in the clinic. In our validation work, we have tested a range of pharmacological manipulations but also used more ethological methods such as social stress and social enrichment. Validation of the ABT is now extensive, including studies in different rat strains and male and female animals [56,78] and suggests very good predictive and translational validity. Further work to develop a human version of the task is needed to help with understanding whether the task also has face and construct validity. The studies involving the neural circuit analysis point in a promising direction and suggest similar brain regions are involved in modulating these behaviours in the ABT. However, interpretation biases in humans have been shown to be sensitive to acute antidepressant treatments, which we and others have not seen in the JBT. This suggests that these behaviours involve different neuropsychological processes. This may arise from the use of cues with innate emotional associations for the human tasks versus cues where the association is first learnt by the rats. The intermediate cue used for the rat studies is either an intermediate tone frequency or spatial position, which is also different from the morphed images most often used for the emotional interpretation tasks. Given that the response of animals in the JBT shifts in the predicted direction following chronic treatment, we suggest that the JBT may involve decision-making where the choice of response is influenced by the learned association between the cue and outcome. Chronic exposure of the animals to the cues while also receiving treatment may enable these biases to slowly develop as the learnt associations are altered and start to influence decision-making behaviour. This could align with the Harmer model where the objective changes in emotional interpretation do not impact on the subjective experience of mood until sufficient new learning has occurred under the influence of the antidepressant treatment [54].

Overall the findings for these two tasks suggest that the affective state of an animal can lead to biases in cognition when we look in the domains of learning and memory and decision-making. The results from the antidepressant studies have revealed some interesting differences. These may be specific to the rodent work but could reveal new insights into how affective states modulate different cognitive domains with distinct time courses.

## 5. Is there a link between affective biases and anhedonia in major depressive disorder?

**Figure 3.**
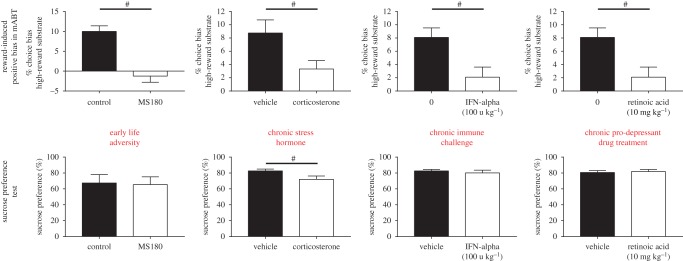
Summary of results for the modified ABT (mABT) showing reward-induced positive bias and its attenuation in putative models of depression. Animals exposed to early life adversity, chronic treatment with corticosterone, interferon alpha or retinoic acid all show impairments in their ability to learn the reward value and fail to bias their choices based on the higher value reward. In contrast to this anticipatory anhedonia, deficits in sucrose preference were only observed in animals receiving the chronic corticosterone treatment. ([75]; SA Stuart and ESJ Robinson 2015, unpublished). (Online version in colour.)

To study affective biases in animals, researchers had to move away from the more typical emotional processing tasks used in human research and instead relied on tasks where novel cues are associated with either rewarding or less rewarding/punishing events. The results from the ABT suggest that memory for rewarding experiences is negatively biased by factors that pose a risk for the development of MDD [56]. The JBT data also show that negative affective states in animals result in a reduced anticipation of reward [61,62,65]. Reductions in reward sensitivity in depression models has been observed previously using the sucrose preference test (SPT) [26], however, results for SPT are not always consistent. Studies using chronic stress or chronic corticosterone treatments generally find deficits in SPT that can be reversed by antidepressants [26,79,80]. In contrast, pro-depressant drug treatments using interferon alpha, rimonabant as well as early life adversity models have not found equivalent deficits [81–83]. There have also been attempts to look at reward learning deficits in rodent tasks designed to recapitulate methods where reward learning deficits have been reported for patients with MDD, e.g. probabilistic learning [60]. However, very little pharmacological or phenotypic data are currently available for these tasks. As discussed above, studies in humans also suggest that the anhedonia in MDD is not related to the ability to experience pleasure but is more about the anticipation of reward, suggesting a more complex, cognitive mechanism. We hypothesized that the effects we observed in the ABT using acute pro-depressant treatments may result in a more sustained deficit in anticipation of reward if experienced chronically. The high- versus low-reward version of the ABT that was used in the initial validation experiments provided a method to test this idea. As shown in figure 3, we have now tested animals that have been treated chronically with pharmacological, environmental and immunological manipulations. In all these groups, we observe a profound deficit in their ability to learn and express a reward-induced positive bias. The findings from the same animals tested using the SPT confirm our hypothesis that this effect is distinct from consummatory anhedonia. Except for chronic corticosterone treatment, which has also previously been shown to impair performance in the SPT, the deficit observed was specific to reward learning in the ABT. In a separate study published by Neill and colleagues, similar impairment in reward-induced positive bias was observed using the ABT in a sub-chronic PCP schizophrenia model [84]. This model is used to study the cognitive and emotional impairments in schizophrenia, although it also fails to show a deficit in the SPT [85]. We propose that this is due to a different underlying neurobiology involving learning and memory of reward-associated events and the ability of the cue associated with reward to re-activate those memories and motivate behaviours accordingly. This is an early finding and more studies are needed to better understand the underlying neurobiology and its relationship to MDD. It will also be important to better understand how the findings in this assay compare with other types of reward learning deficit in MDD. The modified ABT does, however, appear to be able to detect in animals a distinct form of reward deficit that may involve neurobiological processes relevant to the development of anhedonia in MDD.

## 6. Could a neuropsychological mechanism explain the development of major depressive disorder and the efficacy of delayed versus rapid onset antidepressants?

**Figure 4.**
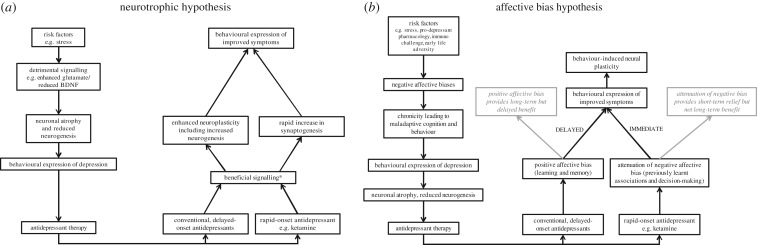
The neurotrophic hypothesis (*a*) and an alternative affective bias hypothesis (*b*), which illustrate the different relationship between behavioural symptoms of MDD and changes in brain morphology. The novel model proposed here reverses the relationship between neuroplasticity and brain atrophy, suggesting these occur because of the behavioural changes induced by negative affective biases. *See [89] for more detailed discussion of relevant signalling pathways. BDNF, brain-derived neurotrophic factor.

One of the most prevalent current theories about the cause of MDD centres around stress-induced detrimental effects on brain morphology causing the behavioural and psychological symptoms of the disease [86–88]. Similarly, the actions of both conventional and rapid onset antidepressants are proposed to arise through an ability to reverse these neuroplastic and neurotrophic deficits, which then leads to the improvement in symptoms [86–88]. An overview of this neurotrophic hypothesis is shown in figure 4*a*. It should also be noted that a recent review by Harmer *et al.* (2017) posed a revised model that is not illustrated here [90]. In their model, the relationship between observations of emotional processing biases and studies in animals showing neuroplasticity changes are discussed, although this model puts neuropsychological and neuroplasticity mechanisms in parallel [90]. Studies in animals certainly suggest that there are detrimental stress-induced changes in brain morphology including reduced neurogenesis and neuronal atrophy [91,92]. Recently, studies with ketamine point towards an NMDA-mediated disinhibition of glutamate release leading to activation of a molecular cascade triggering enhanced brain-derived neurotrophic factor (BDNF) and rapid synapse formation [93,94]. The animal data certainly show that these morphological changes are present but it is still not clear whether they then cause the observed behavioural changes. There is also a lack of evidence supporting direct causality, with studies in animals failing to show the development of a depression-like phenotype when treatments that directly interfere with neurogenesis or neurotrophic factors such as BDNF are tested [95]. Clinical evidence is also limited, with most of the animal work being linked to the finding that hippocampal volume is reduced in patients with MDD [95]. However, early after diagnosis, patients with MDD do not show changes in hippocampal volume and evidence suggests that any reduction in volume correlates with the duration of the disease rather than the severity [96]. Even though the synaptogenesis induced by ketamine is rapid and can occur after a few hours, most studies in patients report behavioural changes almost immediately and suicidal ideation has been shown to change less than one hour after the infusion.

Could an alternative hypothesis be that behavioural changes resulting from negative affective biases lead to the symptoms of MDD? Do the arising maladaptive behavioural consequences of these negative biases then lead to the morphological changes in the brain? This alternative hypothesis is illustrated in figure 4*b*. In this model, risk factors for MDD such as stress or pro-depressant drugs first cause a psychological effect, i.e. negative affective biases. Studies by ourselves and others have shown that animals in putative negative affective states make pessimistic decisions when interpreting ambiguous information linked to cues they have learnt to associate with either positive events or less positive/punishing events. We have also shown that learning and memory associated with reward are effectively devalued by each successive experience the animal encounters in a negative affective state [57]. In the modified ABT, animals in chronic negative affective states fail to appropriately learn reward value (figure 3). These findings predict that chronicity results in these negative biases having detrimental effects on cognition and behaviour, which may then cause morphological changes in the brain. This is speculative but there is evidence that would support a causal relationship between behaviour and changes in hippocampal morphology. For example, studies have observed changes in the volume of the hippocampus of rodents linked to seasonal differences in social and foraging behaviour [97]. Environmental enrichment in laboratory animals increases hippocampal volume [98] and an imaging study in London taxi drivers found evidence of a greater hippocampal volume, which was linked to spatial learning [99]. These may not be directly relevant to MDD but they show that an organism's behaviour and environment can influence the morphology of the brain. In MDD, reduced activity and engagement in rewarding activities and social withdrawal are all well-established characteristics of the disease. It is therefore feasible that these maladaptive behaviours arise from negative affective biases and could in themselves lead to changes in brain morphology.

## 7. Differences in their interaction with affective biases could explain the differential effects of delayed versus rapid onset antidepressants

The actions of antidepressants could be explained by a neuropsychological mechanism involving modification of affective biases that then leads to a normalization of behaviour and the subsequent reversal of the morphological changes (figure 4*b*). Harmer *et al*., [54] previously discussed this idea in terms of emotional processing biases and delayed onset of antidepressants. The animal work suggests that affective biases influence cognition beyond the processing of inherently emotional information. We have shown that conventional antidepressant drugs can enhance the relative reward value attributed to experiences encountered following acute treatment, an effect that can increase with each successive experience. Similarly, conventional antidepressants, when given chronically, cause a gradual shift towards more positive/less negative decision-making in animals, an effect that may be linked to learning and memory. Although not yet tested empirically, the data for the ABT and the effects of conventional antidepressants on learning and memory may directly contribute to the effects seen in the JBT with chronic treatments. The effects of ketamine are particularly interesting in these models since the results for the ABT suggest that ketamine works through its ability to attenuate previously learnt negative biases, an effect that we link to neuronal activity changes in the mPFC as opposed to a neuroplastic effect [57]. Our results find effects following only 30 min pre-treatment and are the same for mPFC infusions of ketamine and the GABA_A_ agonist, muscimol. We also find that ketamine can induce a rapid positive bias in the JBT that we do not observe with the NMDA antagonist, PCP, which is also not an antidepressant in people [100]. In our model, ketamine is only able to modulate negative biases and we failed to see any effects in the ABT in terms of new learning [56], which could explain why its effects are short-term and limited. We hypothesize that ketamine may be acting to neutralize negative biases, enabling patients to shift from a negative affective state to a more neutral state rapidly. This results in a rapid shift in scores in measures of depression and loss of suicidal ideation but they are not being shifted to a positive state. In contrast, conventional antidepressants lack the ability to modify previously acquired negative biases and are therefore delayed in their efficacy because new learning is needed to outweigh the negatively biased memories and develop more positively biased memories and associated behaviours.

## 8. Conclusion

Affective biases offer a plausible neuropsychological explanation for why the symptoms of MDD develop and why delayed versus rapid onset antidepressants differ in their time course of effects. These animal studies suggest that these mechanisms extend beyond emotional processing biases and their impact on social functioning as discussed by Harmer *et al*. [54,90]. They also suggest that there is a direct interaction between the neurochemical effects of these antidepressant treatments and neuropsychological processes that could be used clinically to enhance efficacy. For example, increasing patients' re-engagement in rewarding activities would be a critical component of conventional antidepressant efficacy and the failure of current treatments to work in some populations may reflect their inability to achieve this. Our animal work shows that the symptoms of depression can develop from many different biological causes but these appear to converge on similar neuropsychological processes. Further studies in both clinical populations and animals are now needed to test this hypothesis and better understand the exact details of these relationships. These studies are critical for the development of new treatments and for improving our understanding of how to better use current antidepressants. If neuroplastic and neurotrophic effects are driven by changes in behaviour then these processes are not necessarily going to provide the best drug targets. In terms of both novel neurobiology and the development of new treatments, the ABT and JBT provide useful animal tasks with the potential for better translation to the clinic. It would also be useful to further develop human tasks that work in similar domains so that even closer translation between human and animal work can be achieved.

## Data accessibility

This article has no additional data.

## References

[1] Stuart SA, Butler P, Robinson ES 2014 Animal models of risk factors for suicidal ideation and behaviour. In Suicide: phenomenology and neurobiology, Chapter 18 (eds Cannon KE, Hudzik TJ), pp. 295–314. Cham, Switzerland: Springer International Publishing.

[2] Rumsfeld JS, Nallamothu BK 2008 The hope and fear of rimonabant. JAMA 299, 1601–1602. (10.1001/jama.299.13.1601) 18387935

[3] Bremner JD, McCaffery P 2008 The neurobiology of retinoic acid in affective disorders. Prog. Neuropsychopharmacol. Biol. Psychiatry 32, 315–331. (10.1016/j.pnpbp.2007.07.001) 17707566 PMC2704911

[4] Raison CL, Demetrashvili M, Capuron L, Miller AH 2005 Neuropsychiatric adverse effects of interferon-α: recognition and management. CNS Drugs 19, 105–123. (10.2165/00023210-200519020-00002) 15697325 PMC1255968

[5] Slattery DA, Hudson AL, Nutt DJ 2004 Invited review: the evolution of antidepressant mechanisms. Fundam. Clin. Pharmacol. 18, 1–21. (10.1111/j.1472-8206.2004.00195.x) 14748749

[6] American Psychiatric Association. 2013 Diagnostic and statistical manual of mental disorders, 5th edition Washington, DC: American Psychiatric Association.

[7] Willner P 1984 The validity of animal models of depression. Psychopharmacology (Berl.) 83, 1–16. (10.1007/BF00427414) 6429692

[8] Geyer MAMA 1995 Animal models of psychiatric disorders. In Psychopharmacology: the fourth generation of progress (eds Bloom F, Kupfer D), pp. 787–798. New York, NY: Raven Press.

[9] Belzung C, Lemoine M 2011 Criteria of validity for animal models of psychiatric disorders: focus on anxiety disorders and depression. Biol. Mood Anxiety Disord. 1, 9 (10.1186/2045-5380-1-9) 22738250 PMC3384226

[10] Nestler EJ, Gould E, Manji H 2002 Preclinical models: status of basic research in depression. Biol. Psychiatry 52 503–528. (10.1016/S0006-3223(02)01405-1) 12361666

[11] Willner P 2005 Chronic mild stress (CMS) revisited: consistency and behavioural–neurobiological concordance in the effects of CMS. Neuropsychobiology 52, 90–110. (10.1159/000087097) 16037678

[12] Cryan JF, Holmes A. 2005 The ascent of mouse: advances in modelling human depression and anxiety. Nat. Rev. Drug Discov. 4, 775–790. (10.1038/nrd1825) 16138108

[13] Cryan JF, Slattery DA 2007 Animal models of mood disorders: recent developments. Curr. Opin. Psychiatry 20, 1–7. (10.1097/YCO.0b013e3280117733) 17143074

[14] McArthur RA, Borsini F 2008 PREFACE: What do you mean by ‘translational research’? An enquiry through animal and translational models for CNS drug discovery: psychiatric disorders, in animal and translational models for CNS drug discovery, pp. xvii–xxxviii. San Diego, CA: Academic Press:

[15] Nestler EJ, Hyman SE 2010 Animal models of neuropsychiatric disorders. Nat. Neurosci. 13, 1161–1169. (10.1038/nn.2647) 20877280 PMC3750731

[16] Hales CA, Stuart SA, Anderson MH, Robinson ESJ 2014 Modelling cognitive affective biases in major depressive disorder using rodents. Br. J. Pharmacol. 171, 4524–4538. (10.1111/bph.12603) 24467454 PMC4199314

[17] Berton O, Hahn CG, Thase ME 2012 Are we getting closer to valid translational models for major depression? Science 338, 75–79. (10.1126/science.1222940) 23042886

[18] Slattery DA, Cryan JF 2017 Modelling depression in animals: at the interface of reward and stress pathways. Psychopharmacology (Berl.) 234, 1451–1465. (10.1007/s00213-017-4552-6) 28224183

[19] Commons KG, Cholanians AB, Babb JA, Ehlinger DG 2017 The rodent forced swim test measures stress-coping strategy, not depression-like behavior. ACS Chem. Neurosci. 8, 955–960. (10.1021/acschemneuro.7b00042) 28287253 PMC5518600

[20] Porsolt RD, Le Pichon M, Jalfre M 1977 Depression: a new animal model sensitive to antidepressant treatments. Nature 266, 730–732. (10.1038/266730a0) 559941

[21] Steru L, Chermat R, Thierry B, Simon P 1985 The tail suspension test: a new method for screening antidepressants in mice. Psychopharmacology (Berl.) 85, 367–370. (10.1007/BF00428203) 3923523

[22] Porsolt RD, Bertin A, Blavet N, Deniel M, Jalfre M 1979 Immobility induced by forced swimming in rats: effects of agents which modify central catecholamine and serotonin activity. Eur. J. Pharmacol. 57, 201–210. (10.1016/0014-2999(79)90366-2) 488159

[23] Slattery DA, Cryan JF 2014 The ups and downs of modelling mood disorders in rodents. ILAR J. 55, 297–309. (10.1093/ilar/ilu026) 25225308

[24] Zacharko RM, Anisman H 1991 Stressor-induced anhedonia in the mesocorticolimbic system. Neurosci. Biobehav. Rev. 15, 391–405. (10.1016/S0149-7634(05)80032-6) 1956607

[25] Willner P, Muscat R, Papp M 1992 Chronic mild stress-induced anhedonia: a realistic animal model of depression. Neurosci. Biobehav. Rev. 16, 525–534. (10.1016/S0149-7634(05)80194-0) 1480349

[26] Willner P, Towell A, Sampson D, Sophokleous S, Muscat R 1987 Reduction of sucrose preference by chronic unpredictable mild stress, and its restoration by a tricyclic antidepressant. Psychopharmacology (Berl.) 93, 358–364. (10.1007/BF00187257) 3124165

[27] Dichter GS, Smoski MJ, Kampov-Polevoy AB, Gallop R, Garbutt JC. 2010 Unipolar depression does not moderate responses to the sweet taste test. Depress Anxiety 27, 859–863. (10.1002/da.20690) 20336799 PMC2935496

[28] Sherdell L, Waugh CE, Gotlib IH 2012 Anticipatory pleasure predicts motivation for reward in major depression. J. Abnorm. Psychol. 121, 51–60. (10.1037/a0024945) 21842963 PMC3335300

[29] Ramaker MJ, Dulawa SC 2017 Identifying fast-onset antidepressants using rodent models. Mol. Psychiatry 22, 656–665. (10.1038/mp.2017.36) 28322276

[30] Dulawa SC, Hen R 2005 Recent advances in animal models of chronic antidepressant effects: the novelty-induced hypophagia test. Neurosci. Biobehav. Rev. 29, 771–783. (10.1016/j.neubiorev.2005.03.017) 15890403

[31] Robinson ES, Roiser JP 2016 Affective biases in humans and animals. Curr. Top. Behav. Neurosci. 28, 263–286. (10.1007/7854_2015_5011) 27660073

[32] Gur RC, Erwin RJ, Gur RE, Zwil AS, Heimberg C, Kraemer HC 1992 Facial emotion discrimination: II. Behavioral findings in depression. Psychiatry Res. 42, 241–251. (10.1016/0165-1781(92)90116-K) 1496056

[33] Williams JM, Barnhofer T, Crane C, Herman D, Raes F, Watkins E, Dalgleish T 2007 Autobiographical memory specificity and emotional disorder. Psychol. Bull. 133, 122–148. (10.1037/0033-2909.133.1.122) 17201573 PMC2834574

[34] Surguladze S, Brammer MJ, Keedwell P, Giampietro V, Young AW, Travis MJ, Williams SC, Phillips ML 2005 A differential pattern of neural response toward sad versus happy facial expressions in major depressive disorder. Biol. Psychiatry 57, 201–209. (10.1016/j.biopsych.2004.10.028) 15691520

[35] Leppänen J 2006 Emotional information processing in mood disorders: a review of behavioral and neuroimaging findings. Curr. Opin. Psychiatry 19, 34–39. (10.1097/01.yco.0000191500.46411.00) 16612176

[36] Ressler KJ, Mayberg HS 2007 Targeting abnormal neural circuits in mood and anxiety disorders: from the laboratory to the clinic. Nat. Neurosci. 10, 1116–1124. (10.1038/nn1944) 17726478 PMC2444035

[37] Mathews A, MacLeod C 2005 Cognitive vulnerability to emotional disorders. Annu. Rev. Clin. Psychol. 1, 167–195. (10.1146/annurev.clinpsy.1.102803.143916) 17716086

[38] Gotlib IH, Joormann J 2010 Cognition and depression: current status and future directions. Annu. Rev. Clin. Psychol. 6, 285–312. (10.1146/annurev.clinpsy.121208.131305) 20192795 PMC2845726

[39] Elliott R, Zahn R, Deakin JFW, Anderson IM 2011 Affective cognition and its disruption in mood disorders. Neuropsychopharmacology 36, 153–182. (10.1038/npp.2010.77) 20571485 PMC3055516

[40] Roiser JP, Elliott R, Sahakian BJ 2012 Cognitive mechanisms of treatment in depression. Neuropsychopharmacology 37, 117–136. (10.1038/npp.2011.183) 21976044 PMC3238070

[41] Paul ES, Harding EJ, Mendl M 2005 Measuring emotional processes in animals: the utility of a cognitive approach. Neurosci. Biobehav. Rev. 29, 469–491. (10.1016/j.neubiorev.2005.01.002) 15820551

[42] Beck AT 1967 Depression: clinical, experimental, and theoretical aspects. Hoeber Medical Division, NY: Harper & Row.

[43] Beck AT 2008 The evolution of the cognitive model of depression and its neurobiological correlates. Am. J. Psychiatry 165, 969–977. (10.1176/appi.ajp.2008.08050721) 18628348

[44] Bouhuys AL, Bloem GM, Groothuis TG 1995 Induction of depressed and elated mood by music influences the perception of facial emotional expressions in healthy subjects. J. Affect. Disord. 33, 215–226. (10.1016/0165-0327(94)00092-N) 7790675

[45] Hayward G, Goodwin GM, Cowen PJ, Harmer CJ 2005 Low-dose tryptophan depletion in recovered depressed patients induces changes in cognitive processing without depressive symptoms. Biol. Psychiatry 57, 517–524. (10.1016/j.biopsych.2004.11.016) 15737667

[46] Joormann J, Gotlib IH 2006 Is this happiness I see? Biases in the identification of emotional facial expressions in depression and social phobia. J. Abnorm. Psychol. 115, 705–714. (10.1037/0021-843X.115.4.705) 17100528

[47] Joormann J, Gotlib IH 2007 Selective attention to emotional faces following recovery from depression. J. Abnorm. Psychol. 116, 80–85. (10.1037/0021-843X.116.1.80) 17324018

[48] Chan SW, Goodwin GM, Harmer CJ 2007 Highly neurotic never-depressed students have negative biases in information processing. Psychol. Med. 37, 1281–1291. (10.1017/S0033291707000669) 17493298

[49] McCabe SB, Gotlib IH 1995 Selective attention and clinical depression: performance on a deployment-of-attention task. J. Abnorm. Psychol. 104, 241–245. (10.1037/0021-843X.104.1.241) 7897048

[50] Brittlebank AD, Scott J, Williams JM, Ferrier IN 1993 Autobiographical memory in depression: state or trait marker?. Br. J. Psychiatry 162, 118–121. (10.1192/bjp.162.1.118) 8425125

[51] Papadatou-Pastou M, Miskowiak KW, Williams JMG, Harmer CJ, Reinecke A 2012 Acute antidepressant drug administration and autobiographical memory recall: a functional magnetic resonance imaging study. Exp. Clin. Psychopharmacol 20, 364–372. (10.1037/a0027969) 22731734

[52] Harmer CJ, Bhagwagar Z, Perrett DI, Völlm BA, Cowen PJ, Goodwin GM 2003 Acute SSRI administration affects the processing of social cues in healthy volunteers. Neuropsychopharmacology 28, 148–152. (10.1038/sj.npp.1300004) 12496951

[53] Harmer CJ, Hill SA, Taylor MJ, Cowen PJ, Goodwin GM 2003 Toward a neuropsychological theory of antidepressant drug action: increase in positive emotional bias after potentiation of norepinephrine activity. Am. J. Psychiatry 160, 990–992. (10.1176/appi.ajp.160.5.990) 12727705

[54] Harmer CJ, Goodwin GM, Cowen PJ 2009 Why do antidepressants take so long to work? A cognitive neuropsychological model of antidepressant drug action. Br. J. Psychiatry 195, 102–108. (10.1192/bjp.bp.108.051193) 19648538

[55] Pringle A, Browning M, Cowen PJ, Harmer CJ 2011 A cognitive neuropsychological model of antidepressant drug action. Prog. Neuropsychopharmacol. Biol. Psychiatry 35, 1586–1592. (10.1016/j.pnpbp.2010.07.022) 20673783

[56] Stuart SA, Butler P, Munafò MR, Nutt DJ, Robinson ESJ 2013 A translational rodent assay of affective biases in depression and antidepressant therapy. Neuropsychopharmacology 38, 1625–1635. (10.1038/npp.2013.69) 23503126 PMC3717539

[57] Stuart SA, Butler P, Munafò MR, Nutt DJ, Robinson ESJ 2015 Distinct neuropsychological mechanisms may explain delayed- versus rapid-onset antidepressant efficacy. Neuropsychopharmacology 40, 2165–2174. (10.1038/npp.2015.59) 25740288 PMC4487826

[58] Der-Avakian A, Barnes SA, Markou A, Pizzagalli DA 2016 Translational assessment of reward and motivational deficits in psychiatric disorders. Curr. Top. Behav. Neurosci. 28, 231–262. (10.1007/7854_2015_5004) 26873017 PMC4983255

[59] Der-Avakian A, D'Souza MS, Pizzagalli DA, Markou A 2013 Assessment of reward responsiveness in the response bias probabilistic reward task in rats: implications for cross-species translational research. Transl. Psychiatry 3, e297 (10.1038/tp.2013.74) 23982629 PMC3756297

[60] Bari A, Theobald DE, Caprioli D, Mar AC, Aidoo-Micah A, Dalley JW, Robbins TW 2010 Serotonin modulates sensitivity to reward and negative feedback in a probabilistic reversal learning task in rats. Neuropsychopharmacology 35, 1290–1301. (10.1038/npp.2009.233) 20107431 PMC3055347

[61] Harding EJ, Paul ES, Mendl M 2004 Animal behaviour: cognitive bias and affective state. Nature 427, 312 (10.1038/427312a) 14737158

[62] Enkel T, Gholizadeh D, von Bohlen und Halbach O, Sanchis-Segura C, Hurlemann R, Spanagel R, Gass P, Vollmayr B 2010 Ambiguous-cue interpretation is biased under stress- and depression-like states in rats. Neuropsychopharmacology 35, 1008–1015. (10.1038/npp.2009.204) 20043002 PMC3055368

[63] Papciak J, Popik P, Fuchs E, Rygula R 2013 Chronic psychosocial stress makes rats more ‘pessimistic’ in the ambiguous-cue interpretation paradigm. Behav. Brain Res. 256, 305–310. (10.1016/j.bbr.2013.08.036) 23993861

[64] Anderson MH, Munafò MR, Robinson ES 2013 Investigating the psychopharmacology of cognitive affective bias in rats using an affective tone discrimination task. Psychopharmacology (Berl.) 226, 601–613. (10.1007/s00213-012-2932-5) 23239131

[65] Hales CA, Robinson ES, Houghton CJ 2016 Diffusion modelling reveals the decision making processes underlying negative judgement bias in rats. PLoS ONE 11, e0152592 (10.1371/journal.pone.0152592) 27023442 PMC4811525

[66] Rygula R, Papciak J, Popik P 2013 Trait pessimism predicts vulnerability to stress-induced anhedonia in rats. Neuropsychopharmacology 38, 2188–2196. (10.1038/npp.2013.116) 23660704 PMC3773668

[67] Rygula R, Papciak J, Popik P 2014 The effects of acute pharmacological stimulation of the 5-HT, NA and DA systems on the cognitive judgement bias of rats in the ambiguous-cue interpretation paradigm. Eur. Neuropsychopharmacol. 24, 1103–1111. (10.1016/j.euroneuro.2014.01.012) 24503278

[68] Rygula R, Szczech E, Papciak J, Nikiforuk A, Popik P 2014 The effects of cocaine and mazindol on the cognitive judgement bias of rats in the ambiguous-cue interpretation paradigm. Behav. Brain Res. 270, 206–212. (10.1016/j.bbr.2014.05.026) 24859175

[69] Hales CA, Houghton CJ, Robinson ESJ 2017 Behavioural and computational methods reveal differential effects for how delayed and rapid onset antidepressants effect decision making in rats. Eur. Neuropsychopharmacol. 27, 1268–1280. (10.1016/j.euroneuro.2017.09.008) 29100819 PMC5720479

[70] Anderson MH, Hardcastle C, Munafò MR, Robinson ESJ 2012 Evaluation of a novel translational task for assessing emotional biases in different species. Cogn. Affect. Behav. Neurosci. 12, 373–381. (10.3758/s13415-011-0076-4) 22183974

[71] Rzepa E, Fisk J, McCabe C 2017 Blunted neural response to anticipation, effort and consummation of reward and aversion in adolescents with depression symptomatology. J. Psychopharmacol. 31, 303–311. (10.1177/0269881116681416) 28093022

[72] McCabe C 2016 Neural signals of ‘intensity’ but not ‘wanting’ or ‘liking’ of rewards may be trait markers for depression. J. Psychopharmacol. 30, 1020–1027. (10.1177/0269881116653079) 27296275

[73] McCabe C, Cowen PJ, Harmer CJ 2009 Neural representation of reward in recovered depressed patients. Psychopharmacology (Berl.) 205, 667–677. (10.1007/s00213-009-1573-9) 19529923 PMC2718193

[74] Wood CM et al. 2017 Prevalence and influence of cys407* *Grm2* mutation in Hannover-derived Wistar rats: mGlu2 receptor loss links to alcohol intake, risk taking and emotional behaviour. Neuropharmacology 115, 128–138. (10.1016/j.neuropharm.2016.03.020) 26987983

[75] Stuart SA, Wood CM, Robinson ESJ 2017 Using the affective bias test to predict drug-induced negative affect: implications for drug safety. Br. J. Pharmacol. 174, 3200–3210. (10.1111/bph.13972) 28782244 PMC5595760

[76] Mayberg HS, Brannan SK, Tekell JL, Silva JA, Mahurin RK, McGinnis S, Jerabek PA 2000 Regional metabolic effects of fluoxetine in major depression: serial changes and relationship to clinical response. Biol. Psychiatry 48, 830–843. (10.1016/S0006-3223(00)01036-2) 11063978

[77] Hamani C, Mayberg H, Stone S, Laxton A, Haber S, Lozano AM 2011 The subcallosal cingulate gyrus in the context of major depression. Biol. Psychiatry 69, 301–308. (10.1016/j.biopsych.2010.09.034) 21145043

[78] Hinchcliffe JK, Stuart SA, Mendl M, Robinson EMJ 2017 Further validation of the affective bias test for predicting antidepressant and pro-depressant risk: effects of pharmacological and social manipulations in male and female rats. Psychopharmacology (Berl) 234, 3105–3116. (10.1007/s00213-017-4687-5) 28735366 PMC5597685

[79] Gourley SL, Taylor JR 2009 Recapitulation and reversal of a persistent depression-like syndrome in rodents. Curr. Protoc. Neurosci. Chapter 9, Unit 9.32 (10.1002/0471142301.ns0932s49) PMC2774936 19802817

[80] Gregus A, Wintink AJ, Davis AC, Kalynchuk LE 2005 Effect of repeated corticosterone injections and restraint stress on anxiety and depression-like behavior in male rats. Behav. Brain Res. 156, 105–114. (10.1016/j.bbr.2004.05.013) 15474655

[81] Kosel M, Bilkei-Gorzo A, Zawatzky R, Zimmer A, Schlaepfer TE 2011 Pegylated human interferon alpha 2a does not induce depression-associated changes in mice. Psychiatry Res. 185, 243–247. (10.1016/j.psychres.2009.10.012) 20580843

[82] Shalev U, Kafkafi N 2002 Repeated maternal separation does not alter sucrose-reinforced and open-field behaviors. Pharmacol. Biochem. Behav. 73, 115–122. (10.1016/S0091-3057(02)00756-6) 12076730

[83] Griebel G, Stemmelin J, Scatton B 2005 Effects of the cannabinoid CB1 receptor antagonist rimonabant in models of emotional reactivity in rodents. Biol. Psychiatry 57, 261–267. (10.1016/j.biopsych.2004.10.032) 15691527

[84] Sahin C, Doostdar N, Neill JC 2016 Towards the development of improved tests for negative symptoms of schizophrenia in a validated animal model. Behav. Brain Res. 312, 93–101. (10.1016/j.bbr.2016.06.021) 27312268

[85] Jenkins TA, Harte MK, Reynolds GP 2010 Effect of subchronic phencyclidine administration on sucrose preference and hippocampal parvalbumin immunoreactivity in the rat. Neurosci. Lett. 471, 144–147. (10.1016/j.neulet.2010.01.028) 20097262

[86] Berton O, Nestler EJ 2006 New approaches to antidepressant drug discovery: beyond monoamines. Nat. Rev. Neurosci. 7, 137–151. (10.1038/nrn1846) 16429123

[87] Duman RS, Monteggia LM 2006 A neurotrophic model for stress-related mood disorders. Biol. Psychiatry 59, 1116–1127. (10.1016/j.biopsych.2006.02.013) 16631126

[88] Phillips C 2017 Brain-derived neurotrophic factor, depression, and physical activity: making the neuroplastic connection. Neural Plast. 2017, 7260130 (10.1155/2017/7260130) 28928987 PMC5591905

[89] Duman RS, Li N 2012 A neurotrophic hypothesis of depression: role of synaptogenesis in the actions of NMDA receptor antagonists. Phil. Trans R. Soc. B 367, 2475–2484. (10.1098/rstb.2011.0357) 22826346 PMC3405673

[90] Harmer CJ, Duman RS, Cowen PJ 2017 How do antidepressants work? New perspectives for refining future treatment approaches. Lancet Psychiatry 4, 409–418. (10.1016/S2215-0366(17)30015-9) 28153641 PMC5410405

[91] Duman RS 2014 Neurobiology of stress, depression, and rapid acting antidepressants: remodeling synaptic connections. Depress Anxiety 31, 291–296. (10.1002/da.22227) 24616149 PMC4432471

[92] Duman RS, Li N 2012 A neurotrophic hypothesis of depression: role of synaptogenesis in the actions of NMDA receptor antagonists. Phil. Trans. R. Soc. B 367, 2475–2484. (10.1098/rstb.2011.0357) 22826346 PMC3405673

[93] Autry AE, Adachi M, Nosyreva E, Na ES, Los MF, Cheng P-f, Kavalali ET 2011 NMDA receptor blockade at rest triggers rapid behavioural antidepressant responses. Nature 475, 91–95. (10.1038/nature10130) 21677641 PMC3172695

[94] Li N, Lee B, Liu R-J, Banasr M, Dwyer JM, Iwata M, Li X-Y, Aghajanian G, Duman RS 2010 mTOR-dependent synapse formation underlies the rapid antidepressant effects of NMDA antagonists. Science 329, 959–964. (10.1126/science.1190287) 20724638 PMC3116441

[95] Groves JO 2007 Is it time to reassess the BDNF hypothesis of depression? Mol. Psychiatry 12, 1079–1088. (10.1038/sj.mp.4002075) 17700574

[96] McKinnon MC, Yucel K, Nazarov A, MacQueen GM. 2009 A meta-analysis examining clinical predictors of hippocampal volume in patients with major depressive disorder. J. Psychiatry Neurosci. 34, 41–54. 19125212 PMC2612082

[97] Burger DK, Saucier JM, Iwaniuk AN, Saucier DM 2013 Seasonal and sex differences in the hippocampus of a wild rodent. Behav. Brain Res. 236, 131–138. (10.1016/j.bbr.2012.08.044) 22974551

[98] Huttenrauch M, Salinas G, Wirths O 2016 Effects of long-term environmental enrichment on anxiety, memory, hippocampal plasticity and overall brain gene expression in C57BL6 mice. Front. Mol. Neurosci. 9, 62 (10.3389/fnmol.2016.00062) 27536216 PMC4971077

[99] Woollett K, Maguire EA 2011 Acquiring ‘the knowledge’ of London's layout drives structural brain changes. Curr. Biol. 21, 2109–2114. (10.1016/j.cub.2011.11.018) 22169537 PMC3268356

[100] Hales CA, Houghton CJ, Robinson ESJ 2017 Behavioural and computational methods reveal differential effects for how delayed and rapid onset antidepressants effect decision making in rats. Eur. Neuropsychopharmacol. 27, 1268–1280. (10.1016/j.euroneuro.2017.09.008) 29100819 PMC5720479

